# Protein secretion in human mammary epithelial cells following HER1 receptor activation: influence of HER2 and HER3 expression

**DOI:** 10.1186/1471-2407-11-69

**Published:** 2011-02-14

**Authors:** Yi Zhang, Rachel M Gonzalez, Richard C Zangar

**Affiliations:** 1 Cell Biology and Biochemistry, Pacific Northwest National Laboratory, Richland, WA, 99354, USA; 2 Department of Medicine, University of Tennessee Health Science Center, Memphis, TN, 38163, USA

## Abstract

**Background:**

Protein secretion by mammary cells results in autocrine and paracrine signaling that defines cell growth, migration and the extracellular environment. Even so, we have a limited understanding of the cellular processes that regulate protein secretion.

**Methods:**

In this study, we utilize human epithelial mammary cell (HMEC) lines that were engineered to express different levels of HER1, HER2 and HER3. Using an ELISA microarray platform, we evaluate the effects of epidermal growth factor family receptor (HER) expression on protein secretion in the HMEC lines upon initiation of HER1 receptor activation. The secreted proteins include three HER1 ligands, interleukins 1α and 18, RANTES, vascular-endothelial and platelet-derived growth factors, matrix metalloproteases 1, 2 and 9, and the extracellular portion of the HER1 and HER2 proteins. In addition, we investigate whether MAPK/Erk and PI3K/Akt signaling regulate protein secretion in these cell lines and if so, whether the involvement of HER2 or HER3 receptor alters their response to MAPK/Erk and PI3K/Akt signal pathway inhibition in terms of protein secretion.

**Results:**

Differential expression of HER2 and HER3 receptors alters the secretion of a variety of growth factors, cytokines, and proteases. Some alterations in protein secretion are still observed when MAPK/Erk or PI3K/Akt signaling is inhibited.

**Conclusion:**

This study suggests that HER overexpression orchestrates broad changes in the tumor microenvironment by altering the secretion of a diverse variety of biologically active proteins.

## Background

The family of human epidermal growth factor (EGF) tyrosine kinase receptors (HER) includes HER1 (also known as the EGF receptor), HER2, HER3 and HER4. These receptors play important roles in diverse cellular processes, including but not limited to, cell growth, proliferation and migration [1]. Once activated, HER receptors initiate the recruitment of intermediate signaling proteins, which subsequently activate downstream signal cascades that trigger the cellular responses [2]. HER2 receptors lack a ligand-binding domain and HER3 receptors lack intrinsic tyrosine kinase activity [3]. Even so, HER2 and HER3 form dimers with other ligand-bound HER receptors, and thereby participate in signal transduction. Upon ligand binding, HER1 and HER4 are quickly phosphorylated and activated. Receptor activation can result in the release of their cognate ligands, which then act as a positive feedback loop through autocrine/paracrine signaling.

Aberrant HER receptor signaling, either due to overexpression or mutation of one or more HER receptors or due to abnormal production of their ligands, contributes to the development and progression of a broad spectra of human cancers, including breast, colon, lung, ovarian, and head and neck cancers [4-7]. Since portions of these proteins are all released to the extracellular environment, HER receptors and their ligands are not only potential therapeutic targets for the treatment of these cancers, but also potential cancer biomarkers [8-11].

A number of HER ligands have been identified as cancer biomarkers, including EGF, amphiregulin (AREG), heparin-binding EGF-like growth factor (HB-EGF), and transforming growth factor-α (TGF-α) [12-14]. These ligands are tightly associated with HER receptor expression in a variety of cancer types. For example, studies have demonstrated a number of HER ligands are expressed and correlated with expression of HER receptors in breast cancer patients, and high expression of certain HER ligands are related to the biological aggressiveness of the tumors [15]. All of these ligands are initially synthesized as membrane-anchored proteins [3]. Soluble ligands are released through a process called "shedding", which involves proteolytic cleavage on the extracellular side of the transmembrane domain. Shedding is the last step in the secretion of the biologically active ectodomain of the ligands. Similar to HER ligands, HER receptors undergo shedding during both physiological and pathological conditions. In general, this process is thought to represent one of several feedback mechanisms that prevent prolonged receptor activation. Metalloproteases, including the disintegrin and metalloproteases (ADAMs), are recognized as the major mediators of receptor and ligand ectodomain shedding [3,16,17].

Serum concentrations of secreted HER ligands and HER receptors have been investigated rigorously as potential prognostic factors and therapeutic indicators for many cancer types. However, numerous studies suggest that no single protein biomarker assay may have sufficient sensitivity and specificity to be used clinically, especially for early detection. In particular, the tumor microenvironment appears to be a highly regulated system. Its secretome consists of substantial numbers of proteins that are processed through regulated secretory pathways. There is considerable evidence that secretion of these proteins is altered due to a variety of physiological or pathological conditions. Therefore, in addition to HER receptors and ligands, many other groups of circulating proteins have been examined as potential prognostic factors in diagnosis of human cancers.

One such group of proteins is the cytokines [18,19]. In the pathogenesis of carcinogenesis, pro-inflammatory cytokines such as interleukin-1α (IL-1α), tumor necrosis factor (TNFα), and regulated upon activation, normal T cell expressed and secreted (RANTES), can regulate host responses to infection, inflammation and various immune responses. Pro-inflammatory cytokines can also induce expression of adhesion molecules and metalloproteases, both of which are involved in the process of tumor invasion [20]. Besides cytokines, fibrogenic and angiogenic factors, including basic fibroblast growth factor (bFGF) [21], platelet-derived growth factor (PDGF) [22], vascular endothelial growth factor (VEGF) [23], hepatocyte growth factor (HGF) [24], insulin-like growth factor (IGF) [25], are capable of stimulating mitogen signaling pathways and are involved in a wide variety of cellular processes. Although these proteins are not known to directly interact with the HER receptors, it has been demonstrated that most of them can manipulate HER-regulated signal pathways, commonly by transactivation of these receptors [26-28]. Furthermore, increased expression of these proteins in breast cancer is associated with overexpression of HER family members [29,30]. Hence, an evaluation of the relationship between HER expression and cytokines may add valuable information for cancer prognostics.

In regard to potential biomarkers like growth factors and cytokines, there has been a great deal of research on how these proteins alter epithelial cell function and downstream cell-signaling pathways. In contrast, there is very little known about how changes in epithelial cell processes regulate the secretion of these proteins. A better understanding of these cellular mechanisms is needed if we are to gain useful mechanistic insight into tumor biology based on circulating biomarker data. Recent evidence suggests that circulating levels of breast cancer biomarkers vary with stage [31] and with HER2 receptor status [19]. In this study, we use human mammary epithelial cell lines (HMEC) expressing different levels of HER receptors to examine effects on the secretion patterns of these potential biomarkers. HER1-3 are the most commonly studied HER receptors. Therefore, we focused our study on a parental HME cell line with endogenous HER1 expression, as well as derived cell lines that were transfected to overexpress either HER2 or HER3, while still maintaining the basal HER1 expression. Since these HMEC lines were originally derived from normal breast cells, they are not carcinoma cell lines, and are similar to normal mammary epithelial cells in that they require HER1 activation for proper proliferation and migration responses [32]. In this study, we treat cells with a single concentration of EGF to activate HER1, and then examine the effects on protein secretion in all three HMEC lines. Overall, this study provides novel insight into the underlying molecular processes that regulate biomarker secretion and illustrates how HER2 and HER3 co-expression can affect the secretion of a variety of bioactive proteins that are important in breast cancer development.

## Methods

### Materials

EGF (human recombinant) was purchased from Peprotech (Rocky Hill, NJ). Protease inhibitor cocktail III, as well as all signaling pathway inhibitors, including PI3-kinase inhibitors LY 294002 and wortmannin, MEK inhibitors PD 98059 and U0126, were purchased from Calbiochem (La Jolla, CA). All capture and detection antibodies, including the commercial source and catalog numbers, that were used here for the sandwich ELISA protein microarrays have been previously described [33]. All other reagents were from Sigma Chemical Company (St. Louis, MO), unless otherwise indicated.

### Generation of HER Cell Lines

Human mammary epithelial cell line (HMEC) 184A1L5 was graciously provided by Martha Stampfer (Lawrence Berkeley Laboratory, Berkeley, CA) and maintained at 37 °C in 5% CO_2_/air in DFCI-1 medium supplemented with 12.5 ng/ml EGF as described [34,35].

Both HER2 and HER3 expressing cell lines were derived using a retrovirus-based strategy, as described previously [36,37]. Briefly, transfected cells expressing HER2 or HER3 were screened in DFCI-1 medium with the addition of 250 μg/ml G418 (InVitrogen, Carlsbad, CA) or 2 μg/ml puromycin (Sigma, St. Louis, MO), respectively. The abundances of HER receptors on these transfected cells were characterized using flow cytometry, with Alexa-488 conjugated mAb 7C2 against HER2 and phycoerythrin conjugated anti-HER3 antibody (R&D Systems Inc., Minneapolis, MN). Individual clones of retrovirus transfected HMEC were isolated using cloning rings (Fisher Scientific Inc., Pittsburgh, PA). Expression levels of HER receptors were determined by flow cytometry and western blotting.

Three cell lines were examined in this study. They include the parental cell line 184A1L5 expressing endogenous HER1, which is abbreviated as parental "HER1" cell line in this study. The transfected cell line that overexpresses HER2, as well as the basal HER1 receptor, is abbreviated as "HER2" cell line here; the transfected cell line that overexpresses HER3, as well as the basal HER1 receptor expression, is abbreviated as the "HER3" cell line. Levels of the HER receptors in these cell lines were quantified as described before [36,37]. Results from this analysis showed that there are approximately 2x10^5 ^HER1, 3x10^4 ^HER2 and 2x10^3 ^HER3 receptors on each parental HER1 cell, 1.5x10^5 ^HER1, 6x10^5 ^HER2 and 2x10^3 ^HER3 on each HER2 cell, and 2x10^5 ^HER1, 3 10^4 ^HER2 and 2.8 10^4 ^HER3 on each HER3 cell. It has been reported that the well characterized breast cancer cell line SK-BR3 express about 2x10^5 ^HER1, 6x10^5 ^HER2 and 1x10^4 ^HER3 receptors [38,39]. Based on these reports and another evaluation of HER receptor concentrations in breast cancer cells [40], our HER2 or HER3 cell lines express HER2 or HER3, respectively, at levels that are comparable to those found in breast cancer cells that overexpress these receptors.

### Cell Culture and Treatment

Only cells that were less than fifteen passages from the original frozen stock were used in this study. Each cell line was seeded at 3.0x10^5 ^cells per well of 6-well plates (BD Falcon, Franklin Lakes, NJ) in DFCI-1 culture medium, and were allowed to grow to confluence prior to treatment. Before EGF treatment, cultured cells were fasted for 14-18 h in serum-free, DFCI-1 medium that lacked all supplements except 0.1% bovine serum albumin. Cells were then washed twice with buffered saline, pH 7.2-7.4 (PBS), followed by the addition of 1 ml fresh serum-free medium with 12 ng/ml EGF added. Activated cells were incubated at 37 °C for up to 24 h and samples were collected at fixed time points after EGF addition. Immediately prior to harvesting, cells were chilled by placing the culture dishes on ice.

### Sample Collection and Processing

Conditioned medium (CM) from the cultured cells was transferred to microcentrifuge tubes, and centrifuged at 2000 *g *for 5 min at 4 °C in order to remove any particulates or cell debris. An aliquot of each supernatant was then transferred to another tube that contained a 1/10^th ^volume of 1% casein and green fluorescent protein in PBS (Bio-Rad, Hercules, CA), such that the final concentration of the green fluorescent protein was 100 pg/ml. Green fluorescent protein was used as the antigen in an internal calibrant assay based on a sandwich ELISA. The fluorescent signal from the capture antibody in this assay was used for data normalization using a custom bioinformatics program, ProMAT Calibrator, as described below. The cells on these culture plates were then washed twice with cold PBS, and harvested by adding 200µl of lysis buffer (50 mM pH 7.4 HEPES, 150 mM NaCl, 0.5% NP40, 1 mM PMSF, 1 mM Na_3_VO_4 _and 1% protease inhibitor cocktail III). Cell lysates were collected and centrifuged at 18,500 *g *for 10 min. The protein concentration of cell lysates was measured using the Bicinchoninic Acid protein quantitation kit (Sigma, St. Louis, MO) and averaged (± standard deviation) 1.6 ± 0.3 mg/ml. Since 200µl of lysis buffer was added, we estimate that about 0.32 mg of protein was collected from each plate.

In certain experiments, cultured cells were pre-incubated with a single inhibitor for 1 h prior to EGF addition. These inhibitors, and their concentrations, were 20 µM LY 294002, 0.2 µM Wortmannin, 25 µM PD98059, 10 µM U0126. Stock solutions of each reagent were individually prepared in DMSO and the final concentration of DMSO in the culture medium was 0.1% in all treatments, including controls. At least two independent experiments were performed, with five biological replicates (i.e., five cell culture dishes that were individually processed) in each experiment, for all results described here. Samples were stored at -80°C until analysis.

### ELISA Microarray Analysis

Concentrations of individual proteins in CM and cell lysates were quantitatively measured using sandwich enzyme-linked immunosorbent assay (ELISA), as previously described in detail [33]. Briefly, ELISA microarray chips were custom manufactured using aminosilanated, 25x75 mm glass slides (Erie Scientific, Portsmouth, NH) stamped with a hydrophobic barrier that was used to create 16 wells on each slide. The ELISA reagents used in these analyses have been previously evaluated and shown to have no cross-reactivity and to be able to quantitatively detect purified antigens that were spiked into human serum [41]. The capture antibodies were suspended in PBS at concentrations ranging from 0.5 to 1.0 mg/ml. These antibodies were printed using a GeSiM noncontact NanoPlotter NP2 printer (Quantum Analytics, Foster City, CA). Sixteen identical chips were printed on each slide, such that each chip was isolated by a hydrophobic barrier. Each capture antibody and control reagent was printed in quadruplicate (once in each quadrant) on each chip. Successful printing was confirmed using the RedReflect capability on the ScanArray ExpressHT (PerkinElmer, Santa Clara, CA) laser scanner. The printed slides were blocked with 1% casein in PBS at 4 °C and, after washing, stored desiccated and under vacuum at -20°C until use.

In order to generate standard curves for each of the ELISA analyses, a single mixture containing all the antigens was prepared in 0.1% casein in PBS and containing 100 pg/ml green fluorescent protein. This stock solution of the standard mixture was aliquoted and stored at -80°C, and an aliquot was thawed on a daily basis for each ELISA microarray analyses. For this analysis, the standard stock solution was serially diluted 3-fold to create at least 7 dilutions of the standard mixture. Each dilution, and an antigen-free blank, was analyzed on three seperate chips.

Several incubation steps are included in processing the ELISA microarray chips [41]. Washes were performed between each incubation step by submerging the slides in PBS containing 0.05% Tween-20 (PBS-T). Either 20 μl of the standard mixture or an individual, diluted sample was then pipetted onto each of three replicate chips. Samples were then incubated overnight in a closed chamber with saturated humidity and gentle mixing. Slides were then incubated with a cocktail of all detection antibodies in 0.1% casein/PBS buffer for 2 h. The level of biotinylation was then increased using the biotinyltyramide amplification system (Perkin-Elmer). Finally, slides were submerged in 1 μg/ml of Cy3- or Alexa 647-conjugated streptavidin in PBS-T, and incubated for 1 h in dark. Slides were then quickly rinsed with deionized water and dried. These slides were imaged with a ScanArray Express HT laser scanner. ScanArray Express software was used to quantify the spot fluorescence intensity from the scanned images. Spot fluorescent data were then processed and analyzed using Protein Microarray Analysis Tool (ProMAT) and ProMAT Calibrator [42-44], both of which are open-source, freeware programs (available at http://www.pnl.gov/statistics/ProMAT/) that we developed specifically for processing ELISA microarray data. Standard curves were fit to a four-parameter logistic model and used to estimate the individual protein concentrations in each sample.

### RT-PCR Analysis

Quantitative assessment of mRNA expression was performed by real-time RT-PCR. Total RNA was extracted using RNeasy kit (Qiagen, Valencia, CA). The HMECs used in the quantitative RT-PCR analyses were cultured and harvested in parallel with the cells used in the comparable protein experiments in the CM and cell lysates, although the RNA studies were conducted using cells raised in seperate dishes. Complementary DNA were synthesized from total RNA via reverse transcription using ImProm II reagents (Promega, Madison, WI) and oligo-dT priming. The following primers were employed in the quantitative real-time RT-PCR:

cyclophilin A, forward GAGCTGTTGCAGACAAAGTTC and reverse CCCTGGCACATGAATCCTGG;

AREG, forward CGGAGAATGCAAATATATAGAGCAC and reverse CGTTCACCGAAATATTCTTGC;

TGF-α, forward TTGCTGCCACTCAGAAACAG and reverse ATCTGCCACAGTCCACCTG;

RANTES, forward CCTCATTGCTACTGCCCTCT and reverse GGTGTGGTGTCCCGAGGAATA;

IL-1α, forward GCATACGGGTCCTGGCATCTTGTCC and reverse ATGGTGATCTTCTTGCGGCTCTTGC.

PCR reactions were carried out in a Roche Lightcycler II using 20 ng cDNA and FastStart DNA Master^PLUS ^SYBR Green I reagent according to the manufacturer's instructions (Roche Applied Science, Indianapolis, IN). The PCR cycle parameters were set as denaturation at 95 °C for 10 s, annealing at 55 °C for 5 s and elongation at 72 °C for 10 s for 45 cycles. Melting curve analyses were performed from 60 °C to 95 °C in 0.5 °C increments. RNA from individual cell culture plates was prepared for each treatment group in five replicates. Quantitative RT-PCR data for AREG, TGF-α, IL-1α, and RANTES were normalized based on cyclophilin A transcriptional level [45].

### Quantification of Erk/Akt activation

Phosphorylated Erk1 and Akt levels of cell lysates were quantified by conventional ELISA techniques in a 96-well microplate. R&D System's DuoSet IC ELISA kits were used in these measurements, as described before [37]. The manufacture's protocols, including the lysis buffer, were followed in all the ELISA measurements. Before each ELISA assay, protein concentrations of the cell lysates were measured using the Bicinchoninic Acid protein quantitation kit (Sigma, St. Louis, MO).

### Data Analysis

Concentrations of secreted proteins are presented in pg per ml of conditioned medium (CM). The total volume of medium/dish was a constant within and across studies. Concentrations of individual proteins in cell lysates are presented in ng per mg lysate protein. We used total (i.e., accumulated) secreted protein in the temporal studies for comparisons across the cells lines. An alternative approach would have been to use non-stimulated cells as the control, and present the difference between stimulated and non-stimulated cells. This approach was not used because the cells were first fasted overnight in serum-free medium. To continue to deprive the cells of growth factors would have led to cell death during the remaining 24 hr study, and therefore was not a reasonable option. Rather, the approach used allowed us to compare cell lines in parallel and directly demonstrate the involvement of HER2 or HER3 in protein secretion following HER1 activation.

Statistical differences between cell lines following EGF stimulation and/or signaling pathway inhibition were initially determined by analysis of variance, and then delineated using Fisher's protected least significant difference test using StatView 5.0.1 software (SAS Institute). A significance level of 0.05 was used in all cases.

## Results

### Presence of HER2/HER3 Regulates HER Receptor Ligand Shedding in HMEC

In this study, three HMEC lines, each expressing different levels of HER receptors, were stimulated with 12 ng/ml EGF. These cell lines were used to determine if expression of different HER receptors would affect secretion of selected proteins, especially the HER1 ligands that are shed and thereby further activate HER1 signaling by an autocrine process. A proteomics analysis found that the parental HER1 line used here expresses three HER1 ligands: AREG, HB-EGF and TGF-α [46]. Here, we examined the concentration change of these three shed ligands in the CM, together with EGF, which is an exogenous HER1 ligand that was added to the culture medium.

**Figure 1:**
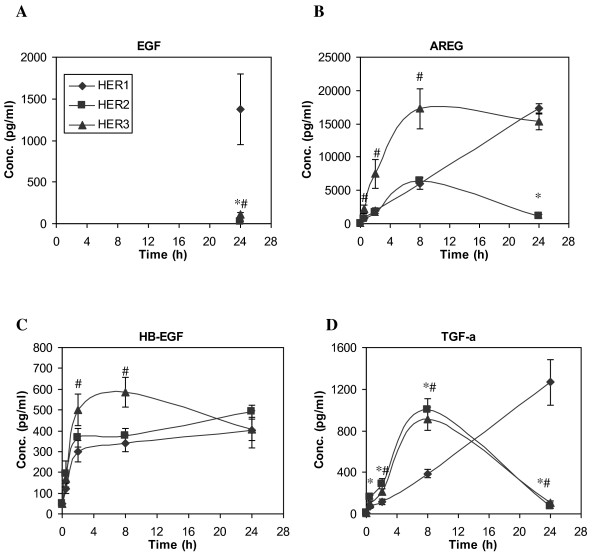
**Time course of HER ligand secretion in HME cell lines expressing different levels of HER receptors after addition of 12 ng/ml EGF to the culture medium**. Conditioned medium (CM) was collected at 0, 0.5, 2, 8 and 24 h after EGF addition, and ELISA microarray analysis was used to determine the concentrations of **A) **EGF, **B) **AREG, **C) **HB-EGF and **D) **TGF-α in CM samples from the parental HER1 cell line (diamonds), HER2 (squares, cell line engineered to overexpress HER2 in addition to the endogenous expression levels of HER1 receptor) and HER3 (triangles, a cell line engineered to overexpress HER3 and has endogenous expression levels of HER1). Except at t = 24 h, EGF concentrations were over the detection limit. Therefore, EGF concentrations at early time points are omitted. Data points and crossbars represent the mean and standard deviation of five replicates, each from a separate culture dish. The symbols denote statistically significant differences between the parental HER1 and HER2 (*) or HER3 (#) by both analysis of variance and Fisher's protected least significant difference analysis.

EGF is the only HER1 ligand that was added to the culture medium, and it has previously been shown not to be synthesized by HMEC cells [46]. We therefore surmise that decreasing EGF concentrations in the CM must reflect EGF consumption. EGF is typically consumed by receptor binding, endocytosis and lysosomal degradation. The initial EGF concentration was 12 ng/ml, which saturates the ELISA analysis. The EGF concentration remained saturating in the CM from the HER1 cells for the first 8 h and dropped to ~1.5 ng/ml at 24 h time point, indicating that EGF was consumed but still remained at the ng/ml level throughout the time course for the parental HER1 cell line. In contrast, the EGF concentration in the CM of HER2 and HER3 cell lines dropped to the pg/ml level at the 24 h time point, indicating a faster EGF consumption rate in these two cell lines (Figure 1A).

Temporal concentration patterns of the other three HER1 ligands, AREG, HB-EGF and TGF-α, are presented in Figure 1B-1D. Rapid and prominent ligand accumulation in the CM was detected in all three cell lines during the first 8 h after initiation of EGF stimulation, with a relatively higher ligand accumulation rate in HER3 cells. Between 8 and 24 h, in most cases, there was no significant or even reduced ligand accumulation, especially in HER2 and HER3 cell lines. Presumably, the attenuation of ligand accumulation in the CM collected from these two cell lines resulted from ligand consumption by HMEC. This seems likely, since ligand consumption would be expected to be accelerated upon the depletion of EGF, which is a competitor for the HER1 receptor and receptor-mediated ligand degradation.

### HER2/HER3 Increase AREG and TGF-α Expression and Shedding

The amount of HER1 ligand shedding can potentially be modulated by either the synthesis of their membrane-anchored protein precursors or the activity of the proteolytic enzymes responsible for their shedding. As differences in ligand shedding was observed between the HMEC lines that express different HER receptors, we examined levels of these ligands in cell lysates. The sandwich ELISA used here should detect the cleaved (i.e., soluble, biologically active) ligand as well as the membrane-bound precursor ligand, which should predominate in the cell lysates.

**Figure 2:**
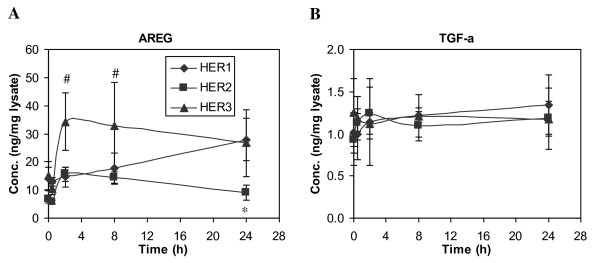
**Time-dependent changes of HER ligand concentrations in cell lysates**. HMEC lines expressing different levels of HER receptors were treated with 12 ng/ml EGF for up to 24 h. Cell lysates were collected at t = 0, 0.5, 2, 8 and 24 h, and ELISA microarray analysis was used to determine the concentration of **A) **AREG and **B) **TGF-α in cell lysates from the parental HER1 cell line (diamonds), HER2 (squares) and HER3 (triangles). Results are presented as ng ligand per mg cell-lysate protein. Data points and crossbars represent the mean and standard deviation of five replicates, each from a separate culture dish. The symbols denote statistically significant differences between the parental HER1 and HER2 (*) or HER3 (#) by both analysis of variance and Fisher's protected least significant difference analysis.

AREG concentrations in cell lysates show the same general pattern as observed for the shed ligand found in the CM (Figure 2A). Even so, AREG concentrations in the cell lysates are significantly greater than those in the media. Therefore, the amount of AREG shedding appears to be primarily dependent on its cellular concentration.

In contrast to AREG, TGF-α concentrations in the cell lysates are relatively constant across all three cell lines (Figure 2B). If the presence of HER2/HER3 does not affect protease activity that is responsible for ligand shedding, similar amounts of shed TGF-α would be predicted. Instead, our results show that there is more accumulation of shed TGF-α in cell lines expressing elevated levels of HER2 or HER3 (Figure 1D). This result suggests that, in contrast to AREG, factors other than just cellular levels of TGF-α influence the process of ligand accumulation in the CM.

### HER2 or HER3 Expression do not Affect AREG and TGF-α mRNA Levels

**Figure 3:**
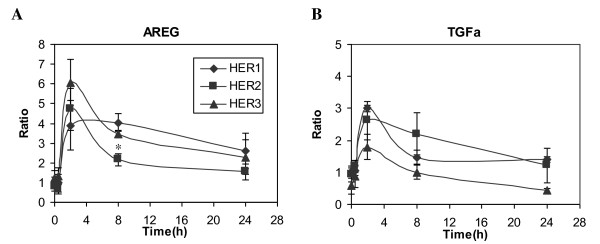
**Analysis of mRNA levels of HER1 ligands**. Relative mRNA levels of **A) **AREG and **B) **TGF-α were determined in HMEC parental HER1 (diamonds), HER2 (squares) and HER3 (triangles) cell lines using quantitative RT-PCR, as described in the Methods. Total RNA was extracted from cell samples treated with 12 ng/ml EGF for 0, 0.5, 2, 8 or 24 h. mRNA expression for each gene was normalized with respect to its expression in the parental HER1 cells at t = 0 h. Data points and crossbars represent the mean and standard deviation of three replicates, each from a separate culture dish. The symbols denote statistically significant differences between the parental HER1 and HER2 (*) or HER3 (#) by both analysis of variance and Fisher's protected least significant difference analysis.

Our analysis of ligand concentrations in CM and cell lysate clearly suggests that HER receptors regulate ligand shedding at the level of cellular protein expression. These results are consistent with several lines of evidence that show that the expression of HER ligands increases at both the mRNA and protein levels in many invasive breast tumors, and that ligand mRNA levels in breast cancer cell lines or breast cancer correlate with their respective protein levels [27]. Therefore, we used quantitative RT-PCR analysis to determine if the presence of HER receptors on HMEC regulated HER ligand expression at the mRNA level. Examples of mRNA levels for two ligands, AREG and TGF-α, are shown in Figure 3. Basal levels of AREG mRNA and TGF-α mRNA were similar in both parental (HER1) and HER2 cell lines. The HER3 cell line expressed slightly higher levels of AREG mRNA but lower levels of TGF-α mRNA than the other two cell lines. In all three cell lines, EGF treatment up-regulated the expression of AREG and TGF-α genes within 2 h. Similar expression patterns were observed for both genes across all the three cell lines during the 24 h time course. Since we did not observe significant differences in mRNA levels for these ligands between the three cell lines, we concluded that the presence of HER2 or HER3 in the HMEC did not significantly alter the pathways that regulate AREG and TGF-α expression upon activation of HER1. These results imply that the production rates of both AREG and TGF-α are similar in all the three cell lines, regardless of the presence of elevated levels of HER2 or HER3. This conclusion further implies that the differences in HER1 ligand concentrations in CM and cell lysates that we observed in these cell lines mainly result from ligand secretion and trafficking and, potentially, subsequent degradation.

### Accumulation of shed HER Ligands is Mediated by Both MAPK/Erk and PI3K/Akt Pathways

**Figure 4:**
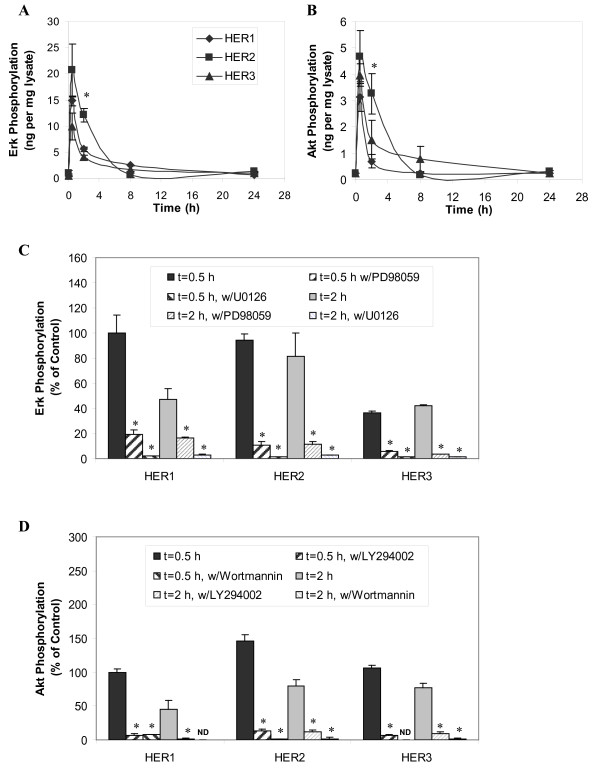
**Erk and Akt activation in response to EGF stimulation in HMEC**. Phosphorylation levels of **A) **Erk and **B) **Akt were examined in HMEC parental HER1 cell (diamonds), HER2 (squares) and HER3 (triangles) using a commercial 96-well-plate ELISA. Lysate sample were collected from cells treated with 12 ng/ml EGF for 0, 0.5, 2, 8 and 24 h. Results are presented as ng pErk or pAkt per mg lysate protein. Phosphorylation level of **C) **Erk and **D) **Akt were also examined when cells were pretreated with MAPK inhibitors (25 µM PD98059 and 10 µM U0126) and PI3K inhibitors (20 µM LY94002 and 200 nM wortmannin). Data points (or columns) and cross bars represent the average and standard deviation, respectively, of two biological replicates, each from a separate culture dish. In panels A and B, the symbols denote statistically significant differences between the parental HER1 and HER2 (*) or HER3 (#) by t-test. In panels C and D, results were normalized based on the phosphorylation level of the control sample (0.1% DMSO alone) from the parental HER1 cells after 0.5 h EGF stimulation. In the presence of wortmannin, Akt phosphorylation levels of parental HER1 cell line at t = 2 hr and HER3 cell line at t = 0.5 hr were below assay detection limit. The asterisks in panel C and D denote statistically significant differences between inhibitor-treated sample and its control (0.1% DMSO in absence of any inhibitor) in each individual cell line at every time point.

**Figure 5:**
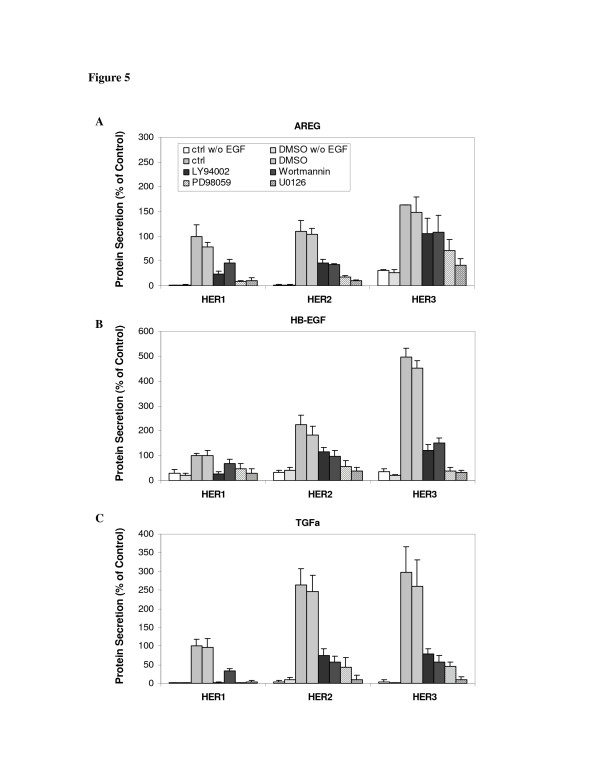
**Effect of MAPK/Erk and PI3K/Akt pathway inhibitors on HER1 ligand secretion**. HMEC lines expressing different levels of HER receptors were preincubated with a single inhibitor (20 μg/ml LY 294002, 200 nM wortmannin, 25 µM PD 98059 or 10 µM U0126) for 1 h, followed by treatment with 12 ng/ml EGF for 8 h. DMSO concentration was 0.1% in all experiments except those marked as "ctrl". Conditioned medium (CM) was collected and the concentrations of **A) **AREG, **B) **HB-EGF and **C) **TGF-α were determined by ELISA microarray analysis. Ligand concentrations were normalized with respect to the concentration in the HER1 cells treated only with 12 ng/ml EGF for 8 h. Columns and crossbars represent the mean and standard deviation, respectively, from five replicates, each of which was from a separate culture dish. The standard deviation is shown for every analysis, but in some cases the crossbar may be too small to be visible. The asterisks denote statistically significant differences between inhibitor-treated cells and ctrl cells for the individual cell lines, as determined by both analysis of variance and by Fisher's protected least significant difference.

HER receptor and ligand autocrine/paracrine signaling has been reported to be regulated by MAPK. Notably, autocrine stimulation of the HER receptor can lead to sustained increases in Erk phosphorylation [47,48]. Furthermore, MAPK kinases also control the proteolytic release of certain HER receptor ligands [49,50]. In this study, we examined Erk and Akt phosphorylation levels in HMEC at 0.5, 2, 8 and 24 h following EGF stimulation. This analysis showed that both phospho-Erk and phospho-Akt signals peaked at ~0.5 h and then gradually dropped to basal level at 8 h (Figure 4A + B). As both MAPK/Erk and PI3K/Akt are key pathways activated by HER1 signaling, we used chemical inhibitors to independently block each of these signal pathways, with the goal of determining their roles in HER1-dependent ligand shedding in the HMEC lines. We observed that two MAPK inhibitors (25 µM PD98059 and 10 µM U0126) and two PI3K inhibitors (20 µM LY94002 and 200 nM wortmannin) could block more than 80% of Erk and Akt activation induced by EGF stimulation, respectively (Figure 4C + D). In addition, each of these inhibitors significantly reduced accumulation of the shed ligands in the CM of all three cell lines, indicating both pathways support HER ligand secretion (Figure 5).

### HER Receptor Shedding is Detectable, but is at Low Levels in HMEC

**Figure 6:**
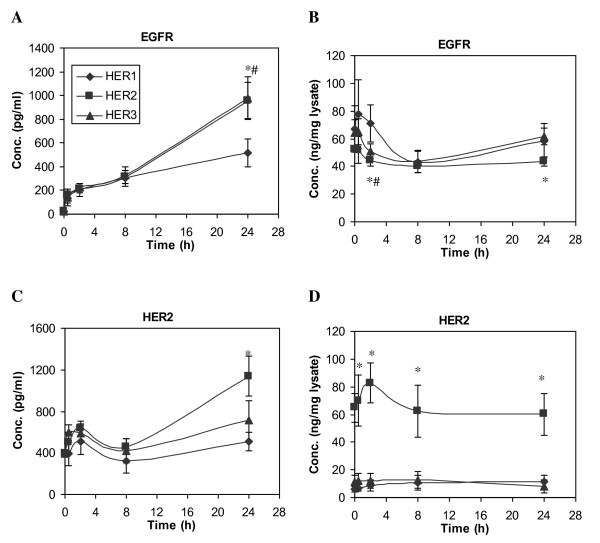
**Time courses of HER receptor concentration change**. HME cell lines expressing different levels of HER receptors were treated with 12 ng/ml EGF for up to 24 h. Both CM and its corresponding cell lysate were collected at t = 0, 0.5, 2, 8 and 24 h. Concentrations of HER1 (EGFR) and HER2 in CM (**A and C**) and cell lysate (**B and D**) were detected from samples of parental HER1 (diamonds), HER2 (squares) and HER3 (triangles) cell lines. Error bars represent one standard deviation from the mean of five biological replicates. The asterisks denote statistically significant differences between HER1 and HER2 (*) or HER3 (#) by both analysis of variance and Fisher's protected least significant difference analysis.

HER receptors undergo shedding in a similar (i.e., protease-dependent) mechanism as their ligands. In this study, we examined the level of both HER1 and HER2 shedding over a 24 h period after initiation of EGF treatment of each cell line. All three HMEC lines we studied express ~2x10^5 ^HER1 per cell. As shown in Figure 6A, detectable amounts of shed HER1 were observable 8 h after initiation of EGF treatment. This shedding process was not dependent upon EGF treatment, since at 8 h there was no significant difference between samples with or without EGF addition (data not shown). After 8 h, HER1 shedding gradually accelerated, with a faster rate in the HER2 and HER3 cell lines. On the other hand, HER1 concentrations in the cell lysates remained at a relatively constant level during the 24 h time course (Figure 6B). The maximum concentration of HER1 in the CM was 1000 pg/ml at 24 h, suggesting that a total of ~1 ng HER1 was shed from HMEC. Since HER1 concentration in cell lysate is about 50 ng per mg protein of cell lysate and there is about 0.32 mg cell lysate collected from each sample (see Methods), the total amount of HER1 present in cell lysate is approximately 16 ng. Thus, our results show that the total shed HER1 that accumulates over 24 h is approximately 6% of its steady-state cellular level (1 ng in the CM compared to 16 ng in the cell lysate). Similar results were obtained for HER2 shedding in the HER2 cells (Figure 6C + D). Overall, even though HER1 and HER2 receptors are shed at detectable levels, our data suggest that this is not a significant mechanism for the down-regulation of these receptors in the HMECs.

### Matrix Metalloproteinase Secretion is Induced upon HER Activation

**Figure 7:**
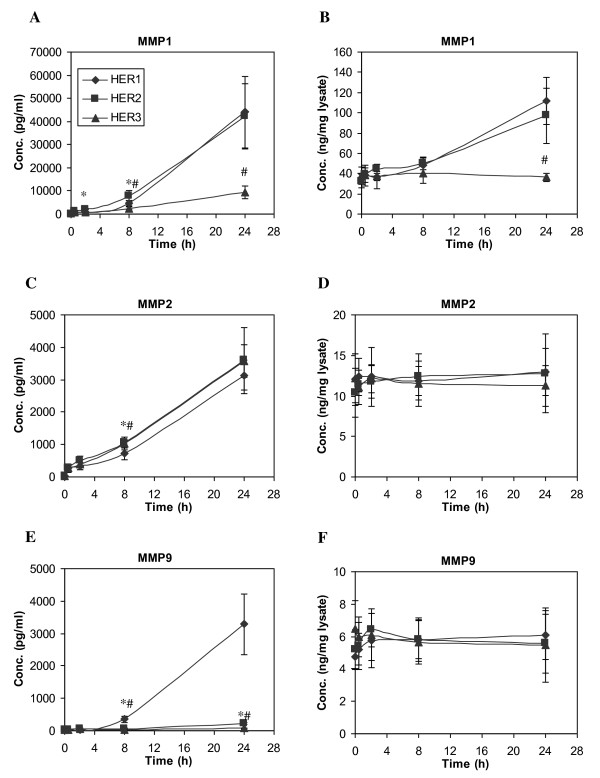
**Time courses of MMP concentration change**. HME cell lines expressing different levels of HER receptors were treated with 12 ng/ml EGF for up to 24 h. Both CM and its corresponding cell lysate were collected at t = 0, 0.5, 2, 8 and 24 h. Concentrations of MMP1, MMP2 and MMP9 in CM (**A, C and E**) and cell lysate (**B, D and F**) were detected from samples of parental HER1 cell line (diamonds), HER2 (squares) and HER3 (triangles). Error bars represent one standard deviation from the mean of five biological replicates. The asterisks denote statistically significant differences between HER1 and HER2 (*) or HER3 (#) by both analysis of variance and Fisher's protected least significant difference analysis.

**Figure 8:**
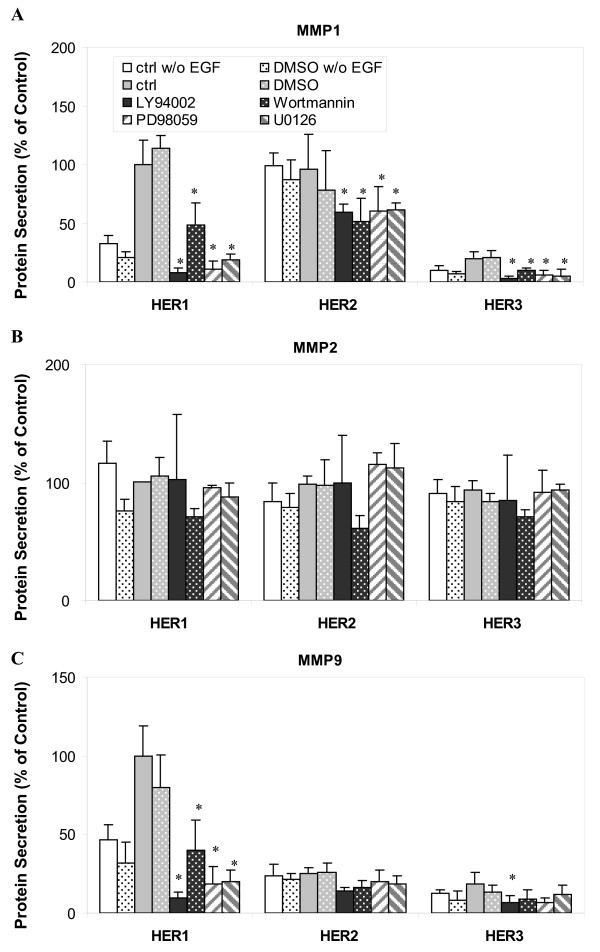
**Effect of MAPK/Erk and PI3K/Akt pathway inhibitors on MMP secretion**. HME cell lines expressing different levels of HER receptors were preincubated with one of the following inhibitors (20 μg/ml LY 294002, 200 nM wortmannin, 25 µM PD 98059 or 10 µM U0126) for 1 h, followed by the treatment with 12 ng/ml EGF for 8 h. DMSO concentration was 0.1% in all the different cell culture media except those marked as "ctrl" runs, which lacked any DMSO. CM was collected and concentration of accumulated **A) **MMP1, **B) **MMP2 and **C) **MMP9 in CM were detected from samples of parental HER1 cell line, HER2 and HER3 cell lines. Each ligand concentrations was normalized with respect to its concentration in the parental HER1 cells that had been treated with 12 ng/ml EGF for 8 h. Error bars represent one standard deviation from the mean of five biological replicates. The asterisks denote statistically significant differences between treated samples and ctrl runs with EGF in each cell line by both analysis of variance and Fisher's protected least significant difference analysis.

The expression and secretion of MMPs are closely associated with cancer cell invasion and metastasis. The proteolytic degradation of extracellular matrix components by MMPs is believed to be required in these metastatic processes [51,52]. MMPs have also been examined as candidate prognostic markers in many types of human cancers [53]. Growth factors have been reported to regulate secretion and activation of various MMPs at both transcriptional level and at the cellular protein level [54]. In this study, we examined the secretion of MMP1, MMP2 and MMP9 in our three HMEC lines following HER1 activation (Figure 7). Our results show that the secretion pattern of MMP2 is not significantly different among the three HMEC lines. Cellular MMP2 concentrations were also similar among the three cell lines. MMP2 secretion is independent of HER1 activation since EGF-treated HMECs release the same amount of MMP2 as untreated cells (Figure 8B). Furthermore, we observe that this constitutive MMP2 secretion is not affected by either MAPK/Erk or PI3K/Akt pathway, since the inhibitors of these pathways have no clear effect on MMP2 secretion (Figure 8B).

Readily detectable levels of secreted MMP1 were observed in all three cell lines. The secretion pattern of MMP1 correlated with its cellular protein concentration (Figure 7A + B), suggesting that MMP1 secretion was regulated by its cellular expression levels. Interestingly, we observed that secretion of MMP1 was regulated by EGF stimulation and controlled by MAPK/Erk and PI3K/Akt pathways in both parental and HER3 cells, but not in HER2 cells (Figure 8A).

In contrast to MMP1, secretion of MMP9 was much higher in the HER1 cells than the HER2 or HER3 cell lines (Figure 7E). MMP9 secretion in the HER1 cells was sensitive to EGF stimulation and both MAPK/Erk and PI3K/Akt inhibitors (Figure 8C). Similar levels of MMP9 were observed in the cell lysates of all three cell lines. Taken together, our results suggest that increased HER2 or HER3 expression attenuates MMP9 secretion induced by HER1 activation.

### Secretion of Various Cytokines and Angiogenic Factors is Triggered by HER Activation

We used the ELISA microarray to analyze four pro-inflammatory cytokines, IL-1α, IL-18, RANTES and TNF-α, and five fibrogenic and angiogenic factors, including bFGF, PDGF, VEGF, HGF and IGF [41]. However, because the concentrations of TNF-α, bFGF, IGF and HGF in CM were essentially undetectable in our assay system, results from these four proteins are not reported here.

**Figure 9:**
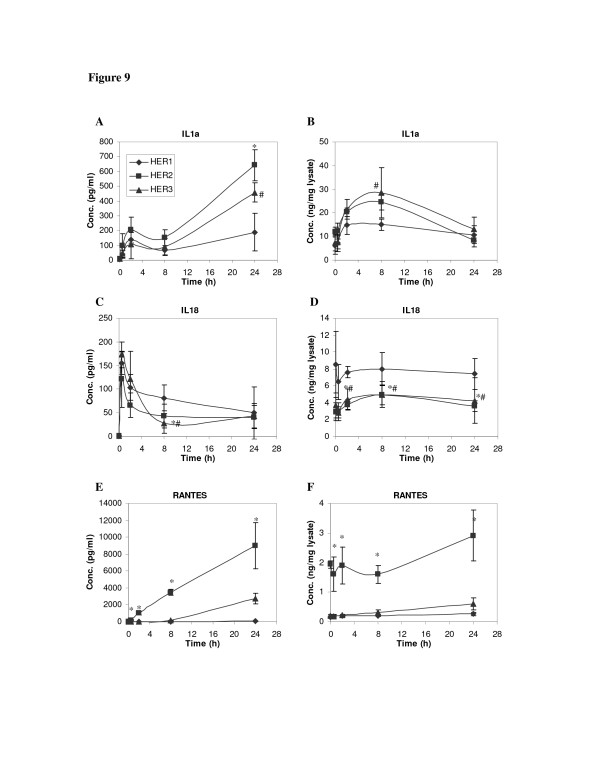
**Time courses of cytokine concentration change**. HME cell lines that express different levels of HER receptors were treated with 12 ng/ml EGF for up to 24 h. Both CM and its corresponding cell lysate were collected. Concentrations of IL-1α, IL-18 and RANTES in CM (**A, C and E**) and cell lysate (**B, D and F**) were detected from samples of parental HER1 cell line (diamonds), HER2 (squares) and HER3 (triangles). Error bars represent one standard deviation from the mean of five biological replicates. The symbols denote statistically significant differences between HER1 and HER2 (*) or HER3 (#) by both analysis of variance and Fisher's protected least significant difference analysis.

**Figure 10:**
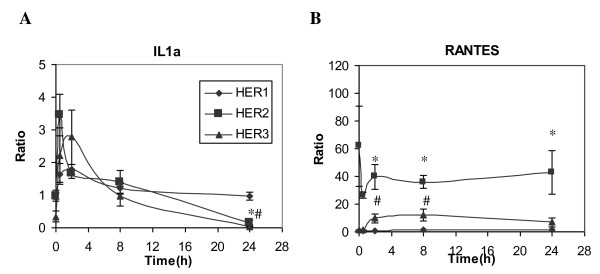
**RT-PCR analysis of IL-1α and RANTES**. Relative mRNA levels of **A) **IL-1α and **B) **RANTES gene expression were examined in HMEC parental HER1 cell (diamonds), HER2 (squares) and HER3 (triangles) using quantitative RT-PCR. Total RNA was extracted from cell samples treated with 12 ng/ml EGF for 0, 0.5, 2, 8 or 24 h. Each mRNA expression level was normalized with respect to its expression in parental cells at t = 0 h. Error bars represent one standard deviation from the mean of three biological replicates. The symbols denote statistically significant differences between HER1 and HER2 (*) or HER3 (#) by both analysis of variance and Fisher's protected least significant difference analysis.

For the three detectable pro-inflammatory cytokines, HER expression levels had qualitatively similar effects on protein concentrations in CM and in cell lysates (Figure 9). For example, RANTES concentration was significantly higher in both CM and cell lysates of HER2 cells than in the other two cell lines. Similar results were observed for RANTES mRNA levels (Figure 10B). Similar to the HER1 ligands, these results suggest that secretion of IL-1α, IL-18 and RANTES are largely dependent upon their cellular protein concentrations in the HMECs.

Our results show that, while IL-18 concentration in CM decreased during the first 0.5 hour of the experiment, both IL-1α and RANTES concentrations continuously increase for the duration of the 24 h experimental period (Figure 9). Although similar temporal patterns of these proteins were observed in all three cell lines, absolute concentrations of these cytokines were consistently greater in the HER2 and HER3 cell lines.

In the cell lysates, IL-1α concentration gradually increased and peaked at 4 to 8 h after initiation of EGF stimulation, particularly in HER2 and HER3 cells (Figure 9B). After 8 h, IL-1α concentrations declined, possibly due to a sustained level of IL-1α secretion and a decreased rate of protein synthesis at the later time. This supposition is supported by the RT-PCR results, which show that IL-1α mRNA expression was suppressed in the HER2 and HER3 cells 24 h after the start of EGF treatment (Figure 10A). Data from our RT-PCR experiments also showed that increases in RANTES mRNA in HER3 were detectable starting at 2 h after EGF treatment (Figure 10B), corresponding to the gradually increasing RANTES concentration in both CM and cell lysates of this cell line (Figure 9E + F).

It should be noted that the CM levels of IL-1α, IL-18 and RANTES after 8 h of EGF treatment were significantly lower than their peak levels at other sampling time points (Figure 9), making it difficult to determine the effect of MAPK and PI3K inhibitors on their secretion at this time point. The only exception was the prominent RANTES concentration in HER2 cell lines (Figure 9E). In the experiment using MAPK and PI3K inhibitors, we did not observe a significant change in the RANTES concentration due to these inhibitors (data not shown), suggesting that secretion of RANTES is independent of both MAPK/Erk and PI3K/Akt pathways.

**Figure 11:**
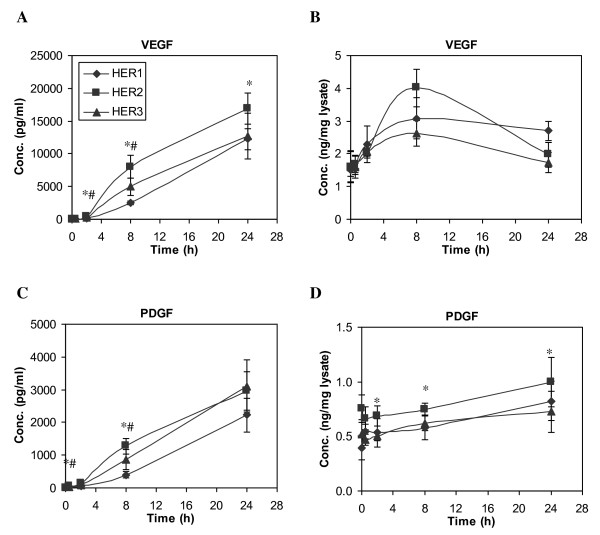
**Time courses of VEGF and PDGF concentration change**. HME cell lines that expressed different levels of HER receptors were treated with 12 ng/ml EGF for up to 24 h. Both CM and its corresponding cell lysate were collected at t = 0, 0.5, 2, 8 and 24 h. Concentrations of VEGF and PDGF in CM (**A and C**) and cell lysate **(B and D) **were measured in samples of parental HER1 (diamonds), HER2 (squares) and HER3 (triangles) cell lines. Error bars represent one standard deviation from the mean of five biological replicates. The symbols denote statistically significant differences between HER1 and HER2 (*) or HER3 (#) by both analysis of variance and Fisher's protected least significant difference analysis.

**Figure 12:**
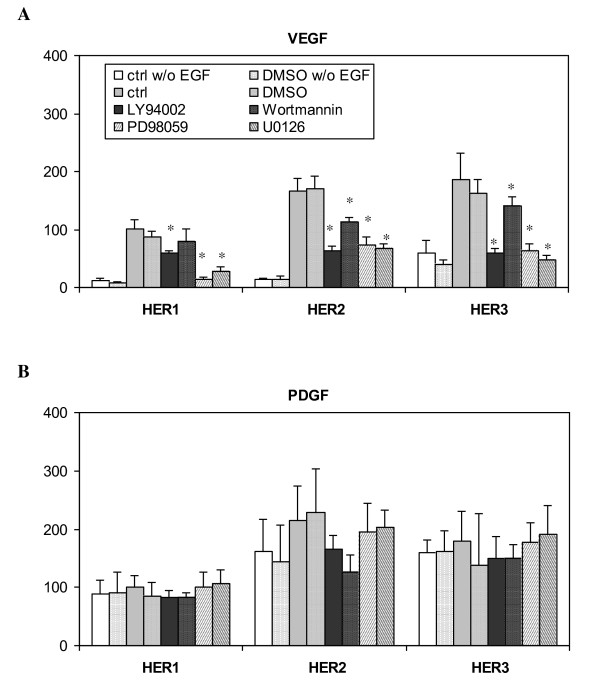
**Effects of MAPK/Erk and PI3K/Akt pathway inhibitors on cytokine secretion in HME cell lines that express different levels of HER receptors**. All cell lines were preincubated with one inhibitor (20 μg/ml LY 294002, 200 nM wortmannin, 25 µM PD 98059 or 10 µM U0126) for 1 h, followed by treatment with 12 ng/ml EGF for 8 h. DMSO concentration in the culture media was 0.1% in all the experiments except those marked as "ctrl" runs, which lacked any DMSO. CM was collected from the parental HER1, HER2 and HER3 cell lines and concentrations of accumulated **A) **VEGF and **B) **PDGF were determined by ELISA microarray analysis. The concentration of each growth factor was normalized with respect to its concentration in the HER1 cells that were treated with 12 ng/ml EGF for 8 h. Error bars represent one standard deviation from the mean of five biological replicates. The asterisks denote statistically significant differences between treated samples and ctrl runs with EGF in each cell line by both analysis of variance and Fisher's protected least significant difference analysis.

Very similar secretion patterns of VEGF and PDGF were observed, in that both proteins continued to accumulate throughout the 24 h experiment (Figure 11A and 11C), with a slightly higher rate in HER2 and HER3 cell lines during the first 8 h. Our results show that secretion of VEGF is a fast and prominent response to EGF stimulation and that this process is mediated through both MAPK/Erk and PI3K/Akt pathways (Figure 12A). A similar concentration pattern of VEGF was observed in the cell lysates, as VEGF concentration increased rapidly in the lysates upon EGF stimulation and peaked at 8 h, with a significantly higher level in HER2 cells. On the other hand, secretion of PDGF does not seem to be a direct response to EGF stimulation, since PDGF concentration in CM at 8 h of EGF stimulation was not significantly different from cells not stimulated by EGF treatment (Figure 12B). These results further indicate that PDGF secretion, at least in this cell context, is not a MAPK/Erk and PI3K/Akt dependent process.

## Discussion

In this study, we examined protein secretion patterns in HMEC upon HER1 receptor activation. This group of proteins not only includes HER receptors and ligands, but a variety of MMPs, cytokines and growth factors that regulate cellular behavior in both normal and pathological conditions. All of these proteins have been associated with the development of a variety of epithelial cancers, including breast cancer; and many of them have been investigated as potential cancer biomarkers. Our goal in this study was to better understand the underlying mechanism that links HER receptor activation and biomarker secretion. In particular, by examining three HMEC lines that express different levels of HER receptors, we attempted to determine the influence of these receptors on the biomarker secretion and their regulatory mechanisms.

Our results suggest that increased HER2 and HER3 expression potentiates ligand induced autocrine and paracrine signaling resulting from EGF activation of HER1. Elevated levels of HER2 or HER3 expression increased HER1 ligand secretion, suggesting an increase in the autocrine positive feedback loop that is associated with HER1 activation (Figure 1). The differences in EGF consumption rate among the three HMEC lines clearly imply that the presence of HER2 or HER3 accelerates HER1 ligand consumption. In contrast to EGF (which is not synthesized by the HMECs), the changes in concentration of AREG, HB-EGF and TGF-α reflect the net balance of two essential processes in this autocrine system: ligand cleavage from the synthesized precursors on cell membrane and ligand binding/consumption through receptor capture and internalization. Because there is a large molar-excess of EGF in the culture medium compared to the secreted HER1 ligands in the early time points of our experiments, it seems likely that the high concentrations of EGF effectively outcompetes the relatively low concentrations of secreted ligands for binding sites on HER1. Thus, the steady accumulation of the secreted HER1 ligands in the CM during those early time points is likely to primarily reflect the rate of ligand shedding. At later time points, when the EGF levels are mostly depleted, the loss of the secreted ligands from the CM likely reflects consumption associated with HER1 binding. All three secreted ligands can contribute to autocrine signaling through HER receptors [32], and all four HER1 ligands assayed here possess similar EC_50 _values for activating HER1 receptor. Even so, ligand binding has been reported to be strengthened when HER1 dimerizes with either HER2 or HER3 [55]. Since, the first step in ligand consumption is binding to the receptor, followed by endocytosis of the receptor-ligand complex and protein degradation, it seems likely that enhancement of HER1 ligand-binding affinities due to increased HER1 dimerization with HER2 or HER3 accounts for the increased EGF consumption observed in the HER2 and HER3 cell lines in the early time points, and increased consumption of other HER ligands at the later time points. It should be noted that, once endocytosed, some HER ligands are not degraded, but they are recycled back to the cell membrane along with HER receptors and are then released from the cell. Compared to HER1, recycling of HER2/HER3 is more prominent [56]. This recycling property potentially may contribute to the increased accumulation of TGF-α in HER2 and HER3 cell lines.

All HER ligands measured in this study can initiate phosphorylation of HER receptors and lead to downstream signal transduction, including the activation of MEK/Erk and PI3K/Akt pathways [1]. Activation of Erk and Akt can provide critical cell mitogenic and survival signals required for tumor progression. Components of these two pathways are frequently abnormal in a variety of cancer tumors and may represent biologically relevant targets for anti-cancer therapy [57-59]. In addition, MEK pathway activation can induce transcriptional activation of HER ligands like TGF-α [59]. Our observation that inhibitors of MAPK/Erk largely blocked HER ligand secretion emphasizes the importance of this pathway in HER autocrine signaling. We also identified a regulatory role of PI3K/Akt in this process (Figure 5). We do not know of any prior reports that identify this pathway in HER1 autocrine signaling. Our study finds that both of these signaling pathways can regulate HER activation by stimulating ligand synthesis and, subsequently, shedding.

In addition to HER ligands, we detected the shedding of HER1 and HER2 in HMEC. Changes in the circulating levels of HER receptors have been reported in a variety of different cancers. Elevated circulating HER2 levels have been identified in a group of patients with HER2-positive breast cancer with significantly worse outcome [60-62]. In contrast, patients diagnosed with ovarian, head and neck, non-small cell lung cancer display lower serum HER1 concentrations, and changes of serum HER1 concentration have been correlated with the efficiency of cancer treatment [11]. In this study, we did not observe prominent HER1 or HER2 shedding in HMEC, even in cells that overexpress HER2 (Figure 6). Our results are consistent with a recent report that investigated HER1 shedding in malignant cells [63]. Their results showed that among a few malignant cell lines, HER1 shedding only occurred in cell lines that express more than 7x10^5 ^HER1 per cell and were treated for 8 h or more with phorbol 12-myristate 13-acetate.

MMPs are widely expressed by many types of cells. High levels of MMPs are associated with tumor invasion and metastasis [54]. Previously, we reported that TNF-α stimulation could induce production of MMP9 in our parental HMEC, the HER1 cell line, and that this process was dependent on autocrine signaling that activated HER1 [64]. In this study, we examined the secreted levels of MMP1, 2 and 9 after EGF treatment. The results show that MMP2 levels are similar among all the three HMEC lines and that EGF treatment does not increase MMP2 levels. Thus, it appears that MMP2 secretion is not regulated by HER receptor activity. In contrast, MMP1 expression and secretion were induced in response to HER signaling. Notably, increased levels of the HER2 receptor enhance basal MMP1 secretion (Figure 8A). In parental cells and HER2 cells, induction of MMP1 expression can be triggered only through ligand-induced HER1 activation. Interestingly, induction of MMP9 secretion by EGF signaling was only observed in parental cells, implying the presence of HER2 or HER3 may repress MMP9 secretion in response to HER1 activation by an undefined mechanism.

Our conclusion that MMP secretion is mediated by the Erk pathway is in agreement with previous reports [65-68]. We also observe that the Akt pathway has a role in MMP secretion (Figure 8). As inhibition of MMP secretion through blockade of either pathway is only effective when secretion is stimulated by HER receptor activation, these results indicate that Erk and Akt pathways mediate MMP secretion through HER signaling in HMEC. Overall, these results suggest that secretion of these three MMP forms are regulated by distinct molecular processes and, thus, that their use as biomarkers may provide differential insight into cell-signaling processes occurring in breast cancer cells.

Cytokines play an important role in the pathogenesis of cancers. Secretion of most cytokines undergo trafficking through the Golgi [69], and are not dependent on proteolytic shedding. In this study, we found that activation of HER signaling could trigger the release of cytokines in the HMEC systems, apparently through increased synthesis of the cytokines (Figure 10).

Continuous accumulation of both PDGF and VEGF was observed in all the HMEC lines. Even so, our results suggest that the regulatory mechanisms that control the secretion of these two proteins are distinct. In HMEC, secretion of PDGF seems to be a constitutive process that is independent of Erk and PI3K/Akt pathways. In contrast, synthesis and secretion of VEGF is a direct and spontaneous response to HER signaling through EGF stimulation, and both MAPK/Erk and PI3K/Akt pathways appear to have a role in VEGF secretion. Our results are consistent with previous studies that demonstrate that HER1 regulates VEGF expression in a variety of different cancer cell lines [70].

## Conclusion

In summary, we examined the interaction between HER receptor levels and protein secretion in HMEC, with the dual goals of gaining insight into the molecular processes that regulate the secretion of proteins that influence cancer development and progression, and therefore may serve as biomarkers that can be used to assess molecular events in tumor tissue. By studying the secretion patterns of a panel of proteins that are associated with a variety of human cancers, we gained novel insight into how HER activation results in a concerted change in multiple classes of secreted proteins that not only alter the cellular microenvironment through matrix reconstruction, angiogenesis, and paracrine interactions with stromal and immune cells, but also provide autocrine feedback to the tumor epithelial cells that results in further enhanced growth and migration. Therefore, this study suggests that changes in protein secretion may be an important factor by which HER activation stimulates mammary tumor formation.

## Competing interests

The authors declare that they have no competing interests.

## Authors' contributions

All authors contributed to the study design, data analysis and approved the final manuscript.

## Pre-publication history

The pre-publication history for this paper can be accessed here:

http://www.biomedcentral.com/1471-2407/11/69/prepub
